# Fungal Secondary Metabolites as Inhibitors of the Ubiquitin–Proteasome System

**DOI:** 10.3390/ijms222413309

**Published:** 2021-12-10

**Authors:** Magdalena Staszczak

**Affiliations:** Department of Biochemistry and Biotechnology, Institute of Biological Sciences, Maria Curie-Skłodowska University, Akademicka 19, 20-033 Lublin, Poland; magdalena.staszczak@poczta.umcs.lublin.pl

**Keywords:** secondary metabolites, natural products, ubiquitin, proteasome, lead compounds, fungi, drug discovery, cancer, ascomycete, basidiomycete

## Abstract

The ubiquitin–proteasome system (UPS) is the major non-lysosomal pathway responsible for regulated degradation of intracellular proteins in eukaryotes. As the principal proteolytic pathway in the cytosol and the nucleus, the UPS serves two main functions: the quality control function (i.e., removal of damaged, misfolded, and functionally incompetent proteins) and a major regulatory function (i.e., targeted degradation of a variety of short-lived regulatory proteins involved in cell cycle control, signal transduction cascades, and regulation of gene expression and metabolic pathways). Aberrations in the UPS are implicated in numerous human pathologies such as cancer, neurodegenerative disorders, autoimmunity, inflammation, or infectious diseases. Therefore, the UPS has become an attractive target for drug discovery and development. For the past two decades, much research has been focused on identifying and developing compounds that target specific components of the UPS. Considerable effort has been devoted to the development of both second-generation proteasome inhibitors and inhibitors of ubiquitinating/deubiquitinating enzymes. With the feature of unique structure and bioactivity, secondary metabolites (natural products) serve as the lead compounds in the development of new therapeutic drugs. This review, for the first time, summarizes fungal secondary metabolites found to act as inhibitors of the UPS components.

## 1. Introduction

The ubiquitin–proteasome system (UPS), also known as the ubiquitin–proteasome pathway (UPP), is the major non-lysosomal system for degrading proteins in eukaryotic cells [1]; it is responsible for the degradation of 80–90% of all cellular proteins [2]. The discovery of ubiquitin (Ub), a ubiquitous and highly conserved protein of 76 amino acid residues, and the understanding of the critical role of the UPS in protein degradation [3,4] was recognized by the 2004 Nobel Prize for Chemistry awarded to Avram Hershko, Aaron Ciechanover, and Irwin Rose. As the principal pathway for degrading cellular proteins, the UPS serves two main functions: (1) a major regulatory function (i.e., targeted degradation of a variety of short-lived regulatory proteins involved in cell cycle control, signal transduction cascades, and regulation of gene expression and metabolic pathways) and (2) the quality control function (i.e., removal of damaged, misfolded, and functionally incompetent proteins).

**Figure 1:**
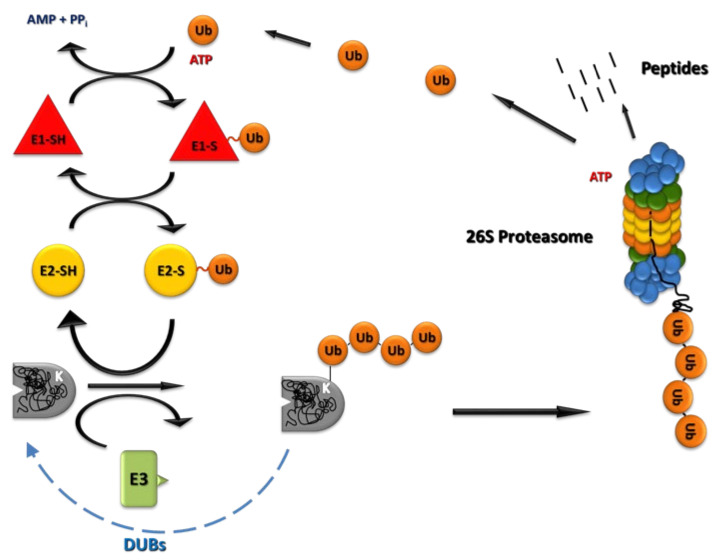
Schematic overview of the ubiquitin–proteasome system (UPS).

The UPS is a highly selective proteolytic system that requires ATP and the 26S proteasome [5,6,7,8], an unusually large (~2.5 MDa) multisubunit complex which degrades protein targets marked by a covalently attached polyubiquitin chain [9,10] (Figure 1). ATP is required both for ubiquitin conjugation to substrates and for the functioning of the 26S proteasome. The presence of the UPS has been demonstrated in animals [11,12,13,14], plants [15,16], and fungi [17,18,19].

Ubiquitin conjugation (ubiquitination or ubiquitylation) is achieved via a hierarchical enzymatic cascade involving the Ub-activating enzyme (E1), Ub conjugating enzymes (E2s), and Ub protein ligases (E3s). The human genome is estimated to encode two E1 isoforms, about 40 E2s, and more than 600 E3 ligases, which generally confer substrate specificity to the UPS [20,21]. Based on their Ub transfer mechanism and molecular structure, E3 enzymes can be subdivided into three major families: RING (Really Interesting New Gene) domain ligases, HECT (homologous to the E6AP carboxyl terminus) domain ligases, and RING-between-RING (RBR) ligases [21,22]. RING domains have a scaffolding role in which they link a catalytically active E2 enzyme to a protein substrate. Ligases containing HECT domains are catalytically active themselves and charged ubiquitin is transferred from an E2 to their catalytic cysteine for direct transfer to a substrate [20]. Typically, multiple ubiquitins linked by the K48-G76 isopeptide bonds form the polyUb chain that is anchored to a lysine residue on the target protein and acts as a signal for recognition [23,24,25] by the 26S proteasome complex located in the cytoplasm and the nucleus [26].

**Figure 2:**
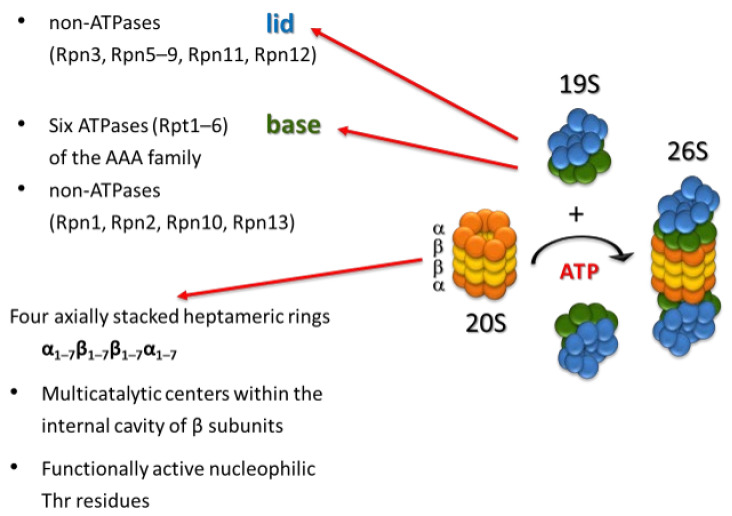
Structure of the 26S proteasome.

**Figure 3:**
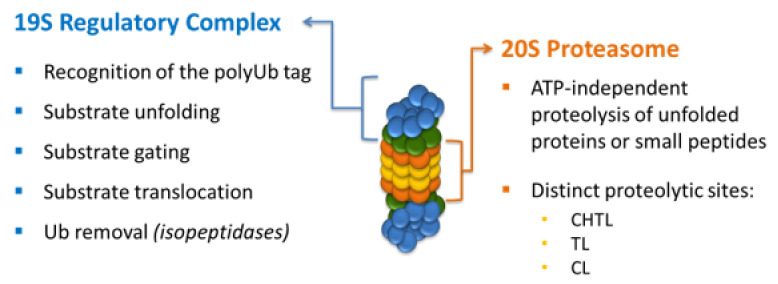
Functions of the 26S proteasome complexes.

The 26S proteasome (Figure 2) is composed of the 20S proteolytic core (20S proteasome or core particle, CP) that is capped at one or both ends by the 19S regulatory complex (regulatory particle, RP) [5,6,7,8]. The 19S regulatory complex confers ubiquitin and ATP dependency to proteolysis, and the 20S proteasome provides the proteolytic activities needed to degrade substrates (Figure 3). The RP is an ~19-subunit complex responsible for recognition and processing of ubiquitinated substrates, and it can be biochemically divided into base and lid subcomplexes [9,27,28,29]. The base is composed of six AAA ATPases (regulatory particle triple-A proteins)—Rpt1–Rpt6 (required for substrate unfolding and translocation into the CP), regulatory particle non-ATPase (Rpn) proteins Rpn1 and Rpn2 (S2 and S1 in humans), and ubiquitin receptors Rpn10 (S5a) and Rpn13. The lid contains nine Rpn subunits (Rpn3, Rpn5–Rpn9, Rpn11, Rpn12, and Rpn15/Sem1 in yeast), including the deubiquitinating enzyme Rpn11, whose activity is essential for efficient substrate degradation [30,31].

After binding to the proteasome, polyubiquitinated protein substrates are unfolded, deubiquitinated, and translocated into the proteolytic core where they are degraded into small peptides. The 20S catalytic core is a cylindrically shaped structure formed by 28 subunits arranged as four stacked heteroheptameric rings (α_1–7_β _1–7_β_1–7_α _1–7_) [25]. The two distal rings are composed of non-proteolytic α-type subunits, while the two inner rings of β-type subunits form the central proteolytic chamber. Each β-ring contains three active sites with distinct substrate specificities. The active site of the β_1_ subunit cleaves after acidic residues (caspase-like, CL), β_2_ cleaves after basic residues (trypsin-like, TL), and β_5_ cleaves after hydrophobic residues (chymotrypsin-like, CHTL). Unlike conventional proteases, all three proteolytic sites in the 20S proteasome utilize their N-terminal threonine residue as the catalytic nucleophile [32,33,34].

An important feature of the UPS is deconjugation of ubiquitin from ubiquitinated substrate proteins through the action of ubiquitin isopeptidases called deubiquitinases (DUBs). There are approximately 90 DUBs encoded in the human genome that are subdivided into six families [35]. Five families are cysteine proteases: ubiquitin-specific proteases (USPs), ubiquitin C-terminal hydrolases (UCHs), ovarian tumor domain proteases (OTUs), Machado–Joseph disease domain proteases (MJDs or Josephins), and motif interacting with ubiquitin-containing novel DUB family (MINDY) DUBs. The sixth class of DUBs, JAB1/MPN/MOV34 (JAMMs), are zinc-dependent metalloproteases (e.g., Rpn11 [30,31]). Catalytic functions of DUBs vary and include the reversal of ubiquitin conjugation, remodeling of ubiquitinated protein substrates, processing of ubiquitin precursors, and cleavage of free polyubiquitin chains to monomeric ubiquitin [36,37,38].

Protein degradation by the ubiquitin–proteasome system plays a crucial role in maintaining cellular proteostasis and regulates key cellular processes, including cell cycle progression, transcription, proliferation, and cell differentiation, apoptosis, signal transduction, morphogenesis, modulation of cell surface receptors and ion channels, antigen presentation, and protein quality control in the endoplasmic reticulum. Dysfunction of the UPS is associated with numerous human pathologies such as cancer, neurodegenerative disorders, autoimmunity, inflammation, or infectious diseases. Therefore, the UPS has become an attractive target for drug discovery and development [39,40,41,42,43,44,45,46,47,48]. For the past two decades, much research has been focused on identifying and developing compounds that target specific components of the UPS. Since the approval of bortezomib/Velcade^®^ [49], the first marketed drug to target proteasomes, considerable effort has been devoted to the development of both second-generation proteasome inhibitors and inhibitors of ubiquitinating/deubiquitinating enzymes [50,51,52,53,54,55,56,57,58,59]. With the feature of unique structure and bioactivity, secondary metabolites of microorganisms and plants serve as the lead compounds in the development of new therapeutic drugs [60,61,62,63,64,65,66,67,68].

Fungi are a rich source of diverse secondary metabolites (SMs), also known as specialized metabolites or natural products [69]. Secondary metabolites are low-molecular-weight natural products with restricted taxonomic distribution, often synthesized after active growth has ceased [70]. In nature, SMs produced by fungi play a crucial role in fungal development, survival, and interactions with other organisms. Some fungal SMs were developed as essential medicines, such as antibiotics (e.g., penicillin) and cholesterol-lowering drugs (e.g., lovastatin). These bioactive compounds are produced by specific fungal taxa, predominately by filamentous fungi from the Pezizomycotina Ascomycota class and several Basidiomycota classes (for example, Agaricomycetes and Exobasidiomycetes) [71]. The actual number of fungal species in existence is estimated at 2.2–3.8 million [72]. In contrast to the genes that are involved in the synthesis of the primary metabolite that are dispersed throughout the fungal genome, the genes encoding the enzymes to produce any secondary metabolites are clustered together in the genome, forming a biosynthetic gene cluster (BGC) [71,73]. The number of BGCs varies across fungi and can range from < 15 to 100 BGCs depending on the fungus [74,75]. BGCs encode for backbone enzymes (also referred to as core enzymes) responsible for creating the core metabolite and tailoring the enzymes that modify this scaffold along with regulatory transcription factors and transporters that transport metabolites and necessary precursors. The backbone enzyme defines the chemical class of the generated secondary metabolite. In fungi, the most common backbone enzymes include non-ribosomal peptide synthetases, polyketide synthases, dimethylallyltransferases, terpene synthases, and terpene cyclases [71,75]. The broad spectrum of biological activities of fungal SMs is a consequence of their structural diversity, which arises from their biosynthetic classification (peptides, polyketides, terpenes, alkaloids) [74].

Re-emerging interest in fungal SMs as the lead structures in drug discovery requires methods to engineer biosynthesis of fungal SMs and activate BGCs that are often silent under the conventional laboratory culture condition [74,76,77]. Significant advances in genome sequencing, bioinformatic tools, natural product chemistry, and increasing ease in fungal genome manipulations have greatly enhanced the ability to efficiently mine fungal genomes for the discovery of new drugs and optimize secondary metabolite production [71,73,74,75,76,77]. Recently, the resistance gene-guided genome mining approach applied to the fungus *Aspergillus nidulans* has revealed that obtaining SMs such as fellutamide B, a proteasome inhibitor, from cryptic SM clusters by serial promoter replacement is feasible and practical and can result in yields that may be commercially useful [78].

This review, for the first time, summarizes the fungal secondary metabolites reported to act as inhibitors of specific components of the UPS. Both inhibitors of the 26S proteasome activities and inhibitors of ubiquitinating/deubiquitinating enzymes are discussed. The inhibitory properties of these compounds, collected from the literature, are listed in Table 1 (proteasome inhibitors), Table 2 (inhibitors of the E1 ubiquitin-activating enzyme), Table 3 (inhibitors of E3 ligases), and Table 4 (inhibitors of deubiquitinases).

## 2. Proteasome Inhibitors

### 2.1. Fellutamides

**Figure 4:**
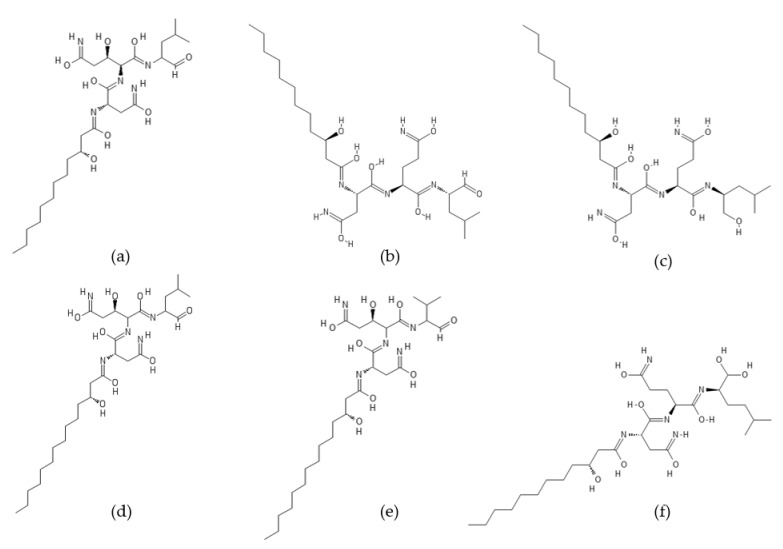
Structures of (**a**) fellutamide A, (**b**) fellutamide B, (**c**) fellutamide C, (**d**) fellutamide D, (**e**) fellutamide E, and (**f**) fellutamide F.

Fellutamides A–F (Figure 4) are a family of cytotoxic lipopeptide metabolites isolated from a range of fungi, including *Penicillium fellutanum* [79], *Aspergillus versicolor* [80,81], *Metulocladosporiella* sp. [82], *Penicillium purpurogenum* G59 [83], and *Aspergillus nidulans* [78]. Chemical structures of fellutamides differ mostly in the length of the hydroxylated fatty acyl chain at the N-terminus and in the side chain group at the C-terminus. Two peptide aldehydes, fellutamides A and B, have been originally isolated from the cultured fungus *P. fellutanum* which is found in the gastrointestinal tract of the marine fish *Apogon endekataenia* [79]. The filamentous fungus *P. fellutanum* has been also isolated from soil samples. A linear lipopeptide structurally similar to fellutamides A and B, named fellutamide C, was initially discovered in *Aspergillus versicolor*, a fungus isolated from a marine sponge *Petrosia* sp. [80]. Two new lipopeptide aldehydes from the soil-derived fungus *Metulocladosporiella* sp. were reported in the same timeframe by Xu et al. [82] and named fellutamides C and D. To avoid ambiguity between the two different compounds named fellutamide C [80,82], fellutamide E [83,84] was proposed as the name of the fellutamide C structure reported by Xu et al. [82]. Fellutamides B and C have also been isolated from an antitumor mutant AD-2-1 obtained by diethyl sulfate (DES) mutagenesis of a marine-derived *Penicillium purpurogenum* G59 [83]. A new cytotoxic lipopeptide named fellutamide F was isolated by bioactivity-guided fractionation from the sponge-derived fungus *A. versicolor* [81].

Fellutamides A–F were discovered during screening for cytotoxic compounds or antifungal agents. Fellutamides A and B were potently cytotoxic against murine leukemia cells (P388 and L1210) and human epidermoid carcinoma KB cells in vitro [79]. In addition, fellutamide B has also been reported to have antiproliferative activity against other cancer cell lines, including sarcoma (S180), fibroblast (NIH 3T3 and LM), glioblastoma (A172), glioma (C6-2B), and pheochromocytoma (PC12) cell lines [85]. Cytotoxic potency of fellutamide C has been shown against human solid tumor cell lines: SK-MEL-2 (skin cancer), XF498 (CNS cancer), and HCT15 (colon cancer) [80]. Antimicrobial activity against fungal pathogens *Candida albicans* and *Aspergillus fumigatus* was reported for fellutamides D and E isolated from *Metulocladosporiella* sp. [82]. In addition, both compounds have shown significant potency against the human prostate carcinoma cells (PC-3) by inducing G2/M cell cycle arrest and apoptosis. Fellutamide F has demonstrated potent cytotoxicity against human skin cancer cells (SK-MEL-2), CNS cancer cells (XF498), and colon cancer cells (HCT15) [81].

Peptide aldehydes like MG132 (Z-Leu-Leu-Leu-al) are known inhibitors of proteasomes [55,86,87]. Fellutamide B, a fungal lipopeptide containing an aldehyde moiety, has been shown to inhibit human proteasomes and induce nerve growth factor (NGF) synthesis [85]. Fellutamide B potently inhibits the chymotrypsin-like activity of the human proteasome with an IC_50_ value of 9.4 ± 2.5 nM. The trypsin-like and caspase-like activities are also inhibited by fellutamide B, but to a lesser extent (IC_50_: 2.0 ± 0.4 µM and 1.2 ± 0.8 µM, respectively). Treatment of LM mouse fibroblasts with fellutamide B led to accumulation of ubiquitinated proteins, confirming its ability to inhibit proteasomes within cells. The crystal structure of the yeast 20S proteasome in complex with fellutamide B has revealed its mechanism of inhibition via formation of a covalent hemiacetal bond between the functional aldehyde group and the Thr1 γ-oxygen at the active site of the proteasome [85]. The peptide backbone of fellutamide B adopts similar conformations, while the long β-hydroxy aliphatic tail assumes different orientations at each of the β_5_, β_2_, and β_1_ catalytic sites. Kinetic studies have revealed that fellutamide B inhibits the human proteasome β_5_ active site following a two-step binding mechanism with *K_i_ =* 11.5 nM and K^*^_i_ = 0.93 nM [88]. Fellutamide B has also been identified as the most potent inhibitor of the β_5_ active site of the *Mycobacterium tuberculosis* (Mtb) proteasome [88]. A single-step binding mechanism with *K_i_* = 6.8 nM has been demonstrated for inhibition of the Mtb proteasome by fellutamide B. Two other lipopeptide aldehydes, fellutamides D and E, were potent in inhibiting the chymotrypsin-like activity of the *C. albicans* proteasome with an IC_50_ value of 0.2 µg/mL (0.33 µM and 0.34 µM, respectively) [82]. Inhibition of fungal proteasomes by fellutamides D and E led to inhibition of fungal growth. Because of the pharmacological potential of natural lipopeptides, total syntheses of fellutamide B as well as of other members of the fellutamide family have been achieved [84,89].

### 2.2. TMC-95A-Related Peptides

**Figure 5:**
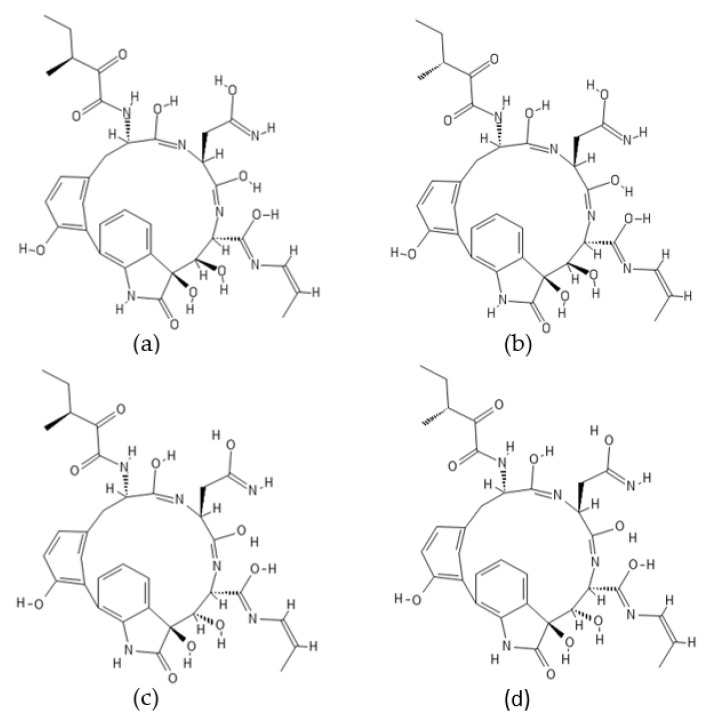
Structures of (**a**) TMC-95A, (**b**) TMC-95B, (**c**) TMC-95C, and (**d**) TMC-95D.

TMC-95A and its diastereomers (Figure 5), TMC-95B, -C, and -D, have been isolated from the fermentation broth of the ascomycete fungus *Apiospora montagnei* Sacc. TC 1093 isolated from a soil sample from a bamboo forest [90,91]. TMC-95s are unusual heterocyclic peptides containing L-tyrosine, L-asparagine, a highly oxidized L-tryptophan, an N-terminal 3-methyl-2-oxopentanoyl moiety, a C-terminal (Z)-1-propenylamine, and a phenol–oxindole ring junction. The molecular formula of TMC-95 macrocyclic peptides has been established as C_33_H_38_N_6_O_10_ [91]. TMC-95s have been identified as potent and selective proteasome inhibitors which competitively and reversibly bind to each of the active β sites (β_5_, β_2_, and β_1_). X-ray crystallographic analysis of the complex of TMC-95A with the yeast 20S proteasome revealed that the inhibitor is bound to the active β subunits by five specific hydrogen bonds and thereby blocks access of the substrate to the catalytic threonine residues [92]. Noncovalent interactions lead to the formation of an anti-parallel β-sheet between the inhibitor and the strictly conserved amino acid residues of the binding pockets. TMC-95A has proven to be the most potent proteasome inhibitor of the TMC-95 family of macrocyclic peptides, inhibiting the chymotrypsin-like, caspase-like, and trypsin-like activity of the 20S proteasome with IC_50_ values of 5.4 nM, 60 nM, and 200 nM, respectively [90]. TMC-95B inhibited these peptidase activities to an extent similar to that of TMC-95A (IC_50_: 8.7 nM (CHTL), 60 nM (CL), and 490 nM (TL)), while TMC-95C and TMC-95D were less effective, inhibiting the chymotrypsin-like, caspase-like, and trypsin-like activities with IC_50_ values of 0.36 µM (TMC-95C) and 0.27 µM (TMC-95D), 8.7 µM (TMC-95C) and 3.3 µM (TMC-95D), and 14 µM (TMC-95C) and 9.3 µM (TMC-95D), respectively. In addition, TMC-95A has shown cytotoxic activities against HCT-116 human colon carcinoma cells and HL60 human promyelocytic leukemia cells with IC_50_ values of 4.4 µM and 9.8 µM, respectively [90].

Noncovalently binding compounds like TMC-95A have potential therapeutic advantages over covalent inhibitors mainly because of their selectivity and weaker side effects. Due to biological activity of TMC-95s, the synthetically challenging TMC-95 core structure has been used for the design and synthesis of simplified analogs, including cyclic peptides [93,94,95], linear tripeptides and dipeptides [96,97,98,99].

### 2.3. Gliotoxin

**Figure 6:**
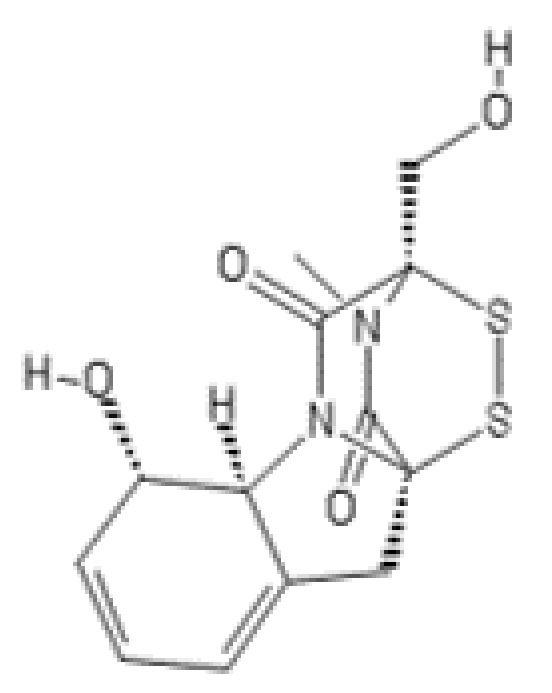
Structure of gliotoxin.

Gliotoxin (Figure 6) is the first reported and the most important epidithiodioxopiperazine (ETP)-type fungal toxin [100,101,102]. This sulfur-containing secondary metabolite (MF: C_13_H_14_N_2_O_4_S_2_) is characterized by an internal disulfide bridge and a diketopiperazine ring that derives from a cyclic dipeptide. Gliotoxin was originally isolated from the wood fungus *Gliocladium fimbriatum* and has since then been identified as an antibiotic produced by various pathogenic species of *Trichoderma* (e.g., *T. virens*)*, Aspergillus* (e.g., *A. fumigatus*), and *Penicillium* [100]. This ETP compound, a known virulence factor in the major human pathogen *A. fumigatus*, has been recently reported to be also biosynthesized by its nonpathogenic relative *A. fischeri* [103].

The toxicity of gliotoxin and other ETP compounds appears to be directly related to the presence of an intact disulfide ring or the reduced dithiol [25]. Gliotoxin has been found to exert cytotoxic effects such as immunosuppression in T and B cells and induction of apoptosis in B cells by inhibiting activation of the transcription factor NF-κB [104]. Since proteasome-mediated degradation of the NF-κB inhibitor IκB is necessary for NF-κB activation, gliotoxin has been suggested to inhibit proteasomal activity [104,105]. Gliotoxin has been demonstrated to act as a noncompetitive inhibitor of the chymotrypsin-like activity of the human 20S proteasome in vitro with an IC_50_ value of approximately 10 µM [105]. The trypsin- and caspase-like activities of the proteasome have been inhibited to a lesser extent. The inhibitory effect of gliotoxin on proteasomes has been attributed to an intact disulfide bridge of the ETP compound [105]. It is worth noting that gliotoxin has also been shown to suppress signal-induced NF-κB activation by selectively inhibiting linear ubiquitin chain assembly complex (LUBAC) [106,107].

Gliotoxin has been found to efficiently target proteasomes in infectious organisms such as the protozoan parasites *Toxoplasma gondii* [108], *Plasmodium falciparum* [109], and *Tritrichomonas foetus* [110]. The progression through the lifecycle of the protozoan parasites requires the functional proteasome. Toxoplasmosis caused by *T. gondii* is one of the most common parasitic infections of humans and other warm-blooded animals. Gliotoxin has been reported to inhibit the growth of *T. gondii* by decreasing the chymotrypsin-like activity of proteasomes [108]. The compound has also been shown to exert plasmodicidal effect on the malaria parasite *P. falciparum* and inhibit the chymotrypsin-like activity of the *P. falciparum* proteasome in a time- and concentration-dependent manner [109].

Gliotoxin at concentrations of 1 and 5 µM inhibited the growth of *T. foetus*, a serious pathogenic parasite of cattle and domestic cats, arresting cell cycle in the G2/M phase and causing DNA fragmentation [110]. The chymotrypsin-like activity of proteasomes isolated from gliotoxin-treated *T. foetus* was inhibited by 48 and 67% at 1 and 5 µM gliotoxin, respectively.

A new assay using the polyubiquitinated protein substrate Ub^n^GST-Wbp2 (WW domain-binding protein 2, *n* > 30) was developed to measure 26S proteasome–mediated protein degradation. This assay has demonstrated that gliotoxin and other ETPs inhibit proteasome activity by targeting the essential deubiquitinase Rpn11 in the 19S regulatory complex of the 26S proteasome [111]. These molecules inhibit Rpn11 activity by chelating the catalytic Zn^2+^ ion (as described below in Section 5.2. Inhibitors of Deubiquitinases).

### 2.4. Epoxyphomalins

**Figure 7:**
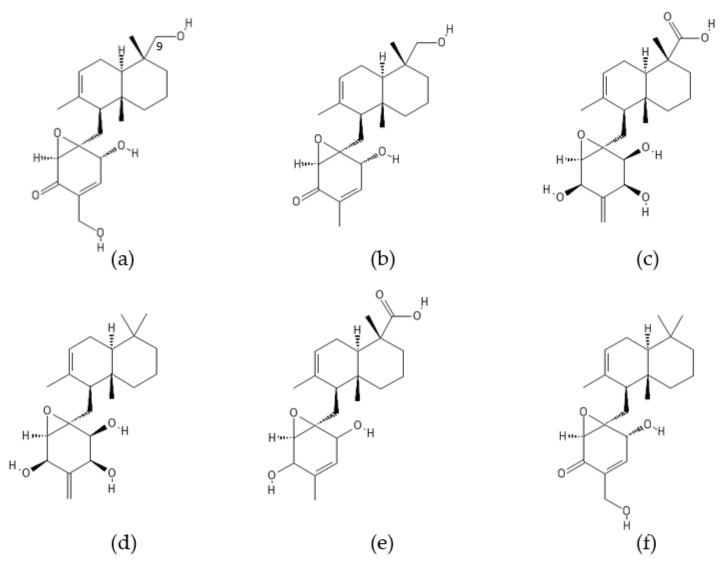
Structures of (**a**) epoxyphomalin A, (**b**) epoxyphomalin B, (**c**) epoxyphomalin C, (**d**) epoxyphomalin D, (**e**) epoxyphomalin E, and (**f**) 11-dehydroxy epoxyphomalin A.

The search for novel anticancer metabolites from filamentous fungi has led to the discovery of two highly cytotoxic sesquiterpenoids [112], named epoxyphomalin A and B (Figure 7) [113,114]. Epoxyphomalins are composed of an isoprenoid-derived decalin ring system linked to a polyketide-derived epoxydon moiety. These structurally unique compounds were isolated from the facultative marine fungus *Phoma* sp. obtained from the Caribbean sponge *Ectyplasia perox* [113,114]. Taxonomy of the fungal strain was reexamined and revised to *Paraconiothyrium* cf *sporulosum* [114]. The molecular formulas of epoxyphomalins A and B were determined to be C_22_H_32_O_5_ and C_22_H_32_O_4_, respectively.

Through bioactivity investigation, using a monolayer cell survival and proliferation assay in a panel of 36 human tumor cell lines, high cytotoxic activity of epoxyphomalins A and B was demonstrated (mean IC_50_ values of 0.11 µg/mL and 1.25 µg/mL, respectively) [113]. Epoxyphomalin A has shown significant tumor cell selectivity toward 12 of the 36 tested tumor cell lines, with IC_50_ values ranging from 0.010 µg/mL (26.6 nM) to 0.038 µg/mL (101 nM) for breast cancer MAXF 401NL and adeno lung cancer LXFA 629 L, respectively. Three new analogs, epoxyphomalins C, D, and E (Figure 7), have also been isolated from *Paraconiothyrium* sp. fermentation extracts and characterized, showing modifications to the oxidation pattern of the cyclohexene moiety or of C-9 of the decalin system [114]. Modest antitumor activity has been observed for epoxyphomalin D (mean IC_50_: 6.12 µM), while epoxyphomalins C and E have not been active at a level of 10 µg/mL (27.6 µM). The new epoxyphomalin analog 11-dehydroxy epoxyphomalin A (Figure 7) has been isolated from the endophytic fungus *Peyronellaea coffeae-arabicae* FT238 obtained from the native Hawaiian plant *Pritchardia lowreyana* [115]. The compound has one hydroxylated methylene and four methyl groups compared to two hydroxylated methylenes and three methyl groups in epoxyphomalin A. It has been demonstrated to show antiproliferative activity against the ovarian cancer cell lines OVCAR3 and A2780 CisR, with IC_50_ values of 0.5 µM and 0.6 µM, respectively [115].

The COMPARE analysis and in vitro enzymatic assays with purified human erythrocyte-derived 20S proteasome have enabled the identification of proteasomes as intracellular targets of epoxyphomalins A and B [114]; both metabolites exhibit dose-dependent inhibition of the chymotrypsin-, trypsin-, and caspase-like proteasome activities. Epoxyphomalin A displays almost equipotent inhibition against all the catalytic sites (β_5_, β_2_, and β_1_), whereas epoxyphomalin B preferentially inhibits the chymotrypsin-like activity. Epoxyphomalins A and B have been therefore proposed to exert their cytotoxic effect through potent inhibition of the 20S proteasome.

### 2.5. Neomacrophorins

**Figure 8:**
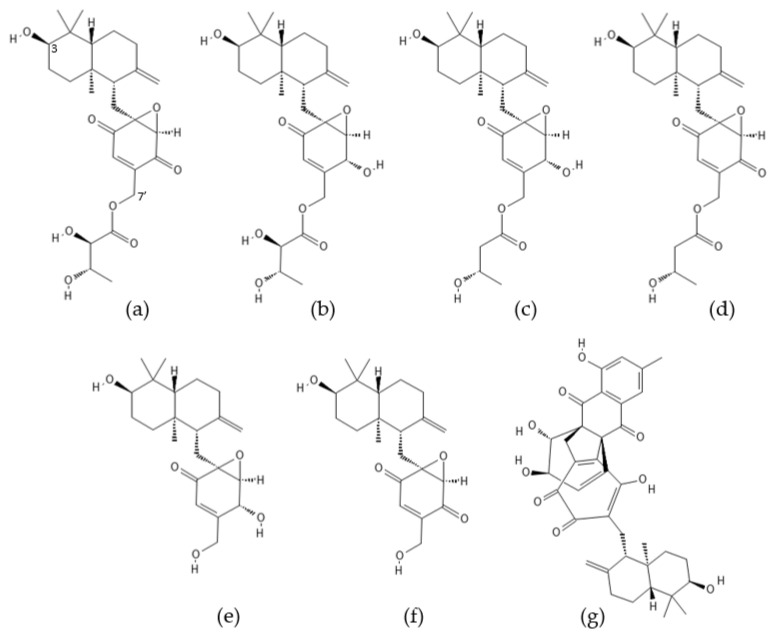
Structures of (**a**) neomacrophorin I, (**b**) neomacrophorin II, (**c**) neomacrophorin III, (**d**) neomacrophorin IV, (**e**) neomacrophorin V, (**f**) neomacrophorin VI, and (**g**) neomacrophorin X.

Neomacrophorins I–VI and X (Figure 8) have been identified as terpenoid metabolites of soil-dwelling *Trichoderma* sp. 1212-03 isolated from the fruit body of the basidiomycete fungus *Daedaleopsis tricolor* in the Shirakami Natural Science Park in 2012 [116,117,118]. Structural analyses of neomacrophorins I, II, and III [116] isolated from the mycoparasitic fungus *Trichoderma* sp. 1212-03 have revealed that these compounds resemble known macrophorins but possess an axial hydroxyl group at C3 as well as different side chains at C7′. Neomacrophorin I has been found to show weak cytotoxicity (IC_50_: 96.6 µM) against human colorectal cancer COLO 201 cells [116]. Neomacrophorin X (MF: C_37_H_40_O_9_), a meroterpenoid [112] that possesses a unique [4.4.3]propellane moiety, has been isolated from the culture broth and the mycelium of *Trichoderma* sp. 1212-03 [117]. The compound has displayed moderate cytotoxicity (IC_50_: 9.3 µM) against human promyelocytic leukemia HL60 cells. Furthermore, neomacrophorin X has been shown to inhibit the human erythrocyte-derived 20S proteasome. The chymotrypsin-like and caspase-like proteasome activities have been inhibited to a similar extent (IC_50_: 18.5 µM and 17.3 µM, respectively), whereas the trypsin-like activity has been less sensitive to neomacrophorin X (IC_50_ >50 µM).

Neomacrophorins I–VI have been demonstrated to inhibit growth of human promyelocytic leukemia HL60 cells with IC_50_ values of 2.6 µM (neomacrophorin I), 21.6 µM (neomacrophorin II), 15.0 µM (neomacrophorin III), 1.3 µM (neomacrophorin IV), 25.3 µM (neomacrophorin V), and 0.3 µM (neomacrophorin VI). The compounds were also responsible for inducing apoptosis in the HL60 cells [118]. Neomacrophorins I (MF: C_26_H_36_O_8_), IV (MF: C_26_H_36_O_7_), and VI (MF: C_22_H_30_O_5_) which contain a quinone moiety have exhibited considerably higher cytotoxicity compared to the other neomacrophorins. Furthermore, neomacrophorins I–IV and VI have shown dose-dependent inhibition of proteasomal activities in vitro. Neomacrophorins I and IV have been found to exhibit the most potent inhibition of the chymotrypsin-like activity (IC_50_: 5.7 ± 1.0 µM and 5.3 ± 1.2 µM, respectively) and the caspase-like activity (IC_50_: 5.2 ± 1.0 µM and 3.9 ± 0.3 µM, respectively) of the human erythrocyte-derived 20S proteasome. The trypsin-like activity has also been inhibited by neomacrophorins I and IV, but to a lesser extent (IC_50_: 27.9 ± 3.3 µM and 22.5 ± 1.3 µM, respectively). The inhibition of proteasomal activities by neomacrophorins has also led to accumulation of ubiquitinated proteins in the HL60 cells. The inhibitory potential of neomacrophorins against proteasomes has been found to coincide with their cytotoxicity and apoptotic effects. The quinone moiety of neomacrophorins I, IV, and VI has been assumed to play an essential role in targeting proteasomes [118].

### 2.6. Trilongins

Trilongins are bioactive peptaibols, secondary metabolites mainly found in the ascomycete fungi *Trichoderma longibrachiatum* (trilongin A and B series) and *T. atroviride* (trilongin C series) [119]. Peptaibols, linear peptides containing 5–20 amino acid residues are synthesized by multidomain megaenzymes named non-ribosomal peptide synthetases. Trilongins have 13 analogs with 11 amino acids (trilongin A series) or 20 amino acids (trilongin B and C series). As other peptaibols, trilongins are characterized by a high content of helix-promoting α-aminoisobutyric acid (Aib), an acetylated N-terminal group, and an amino alcohol, such as phenylalaninol (Pheol) at the C-terminus. Trilongins are mitochondriotoxic to mammalian cells by forming voltage-dependent Na^+^/K^+^-permeable channels in biomembranes [120].

Trilongins BI, BII, BIII, and BIV, originally isolated from the human opportunistic pathogen *T. longibrachiatum*, have been identified as AcAib-Ala-Aib-Ala-Aib-Ala-Gln-Aib-Val/Iva-Aib-Gly-Leu/Ile-Aib-Pro-Val/Iva-Aib-Aib-Gln-Gln-Pheol (trilongin BI), AcAib-Ala-Aib-Ala-Aib-Ala-Gln-Aib-Val/Iva-Aib-Gly-Leu/Ile-Aib-Pro-Val/Iva-Aib-Val/Iva-Gln-Gln-Pheol (trilongin BII), AcAib-Ala-Aib-Ala-Aib-Aib-Gln-Aib-Val/Iva-Aib-Gly-Leu/Ile-Aib-Pro-Val/Iva-Aib-Aib-Gln-Gln-Pheol (trilongin BIII), and AcAib-Ala-Aib-Ala-Aib-Aib-Gln-Aib-Val/Iva-Aib-Gly-Leu/Ile-Aib-Pro-Val/Iva-Aib-Val/Iva-Gln-Gln-Pheol (trilongin BIV) [120]. Trilongins BI, BII, BIII, and BIV with inhibitory activity against the 20S proteasome and antifungal activity against the plant pathogen *Colletotrichum gloeosporioides* (from the guarana plant) have also been isolated from the culture medium of *Trichoderma* sp. P8BDA1F1, an endophytic fungus from *Begonia venosa* [121]. Trilongins BI–BIV have been found to inhibit the chymotrypsin-like activity of the purified 20S yeast proteasome in a dose-dependent manner with IC_50_ values of 6.5 ± 2.7 µM (trilongin BI), 4.7 ± 1.8 µM (trilongin BII), 6.3 ± 2.2 µM (trilongin BIII), and 2.7 ± 0.5 µM (trilongin BIV). Antagonistic interactions with fungal plant pathogens have been found to highly influence the production of trilongins by *Trichoderma* strains [122].

### 2.7. Terrein

**Figure 9:**
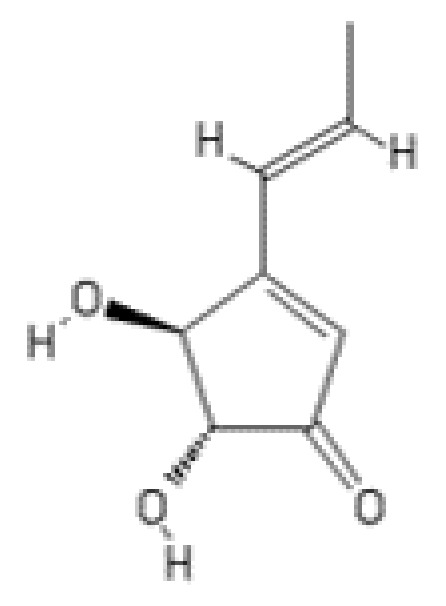
Structure of terrein.

Terrein (4,5-dihydroxy-3-[(E)-1’-propenyl]-2-cyclopenten-l-one, C_8_H_10_O_3_) (Figure 9) is a fungal secondary metabolite originally isolated from *Aspergillus terreus* Thom in 1935 [123]. Terrein has been reported to exhibit various biological activities, including cytotoxicity against human colon cancer COLO205 cells (IC_50_: 50 µM) [124], colorectal carcinoma HCT-116 cells (IC_50_: 12.13 µM), and hepatocellular carcinoma HepG2 cells (IC_50_: 22.53 µM) [125]. The compound has also been found to inhibit cell proliferation and induce G2/M phase cell cycle arrest in human ovarian cancer SKOV3 cells and ovarian cancer stem-like cells [126]. *A. terreus* strain S020 isolated from deep-sea sediment of the Red Sea has been reported to have great potential for industrial production of terrein on a large scale [125]. Synthetic terrein prepared by using the modified Altenhach’s procedure has been shown to inhibit the growth of various head and neck cancer cells [127].

Through the screening of different fungal species (*Aspergillus terreus*, *Aspergillus niger*, *Aspergillus foetidus*, *Rhizopus oryzae*, and *Penicillium decumbens*) for inhibitors of the 20S proteasome, terrein isolated from *A. terreus* cultured in a synthetic medium was found to inhibit proteasomal activity [128]. The cyclopentenone core of terrein and free hydroxyl groups at positions 2 and 3 of the core are assumed to play a crucial role in the inhibition of the 20S proteasome. In vitro enzymatic assays with a purified horse erythrocyte-derived 20S proteasome have demonstrated the ability of terrein to inhibit the chymotrypsin-like and trypsin-like activities with an IC_50_ value of 300 µM. Furthermore, terrein has also inhibited 20S proteasome-catalyzed proteolysis in a dose-dependent manner, as shown by assays with a full-length protein substrate (FITC-casein). The compound has been demonstrated to inhibit the chymotrypsin- and trypsin-like activities in intact fibroblast cells with an IC_50_ value of 75 µM. Terrein has also been found to promote fast apoptotic cell death in NIH-3T3 fibroblast cells and human lung cancer NCI-H292 cells [128].

### 2.8. Pyrrolizilactone

**Figure 10:**
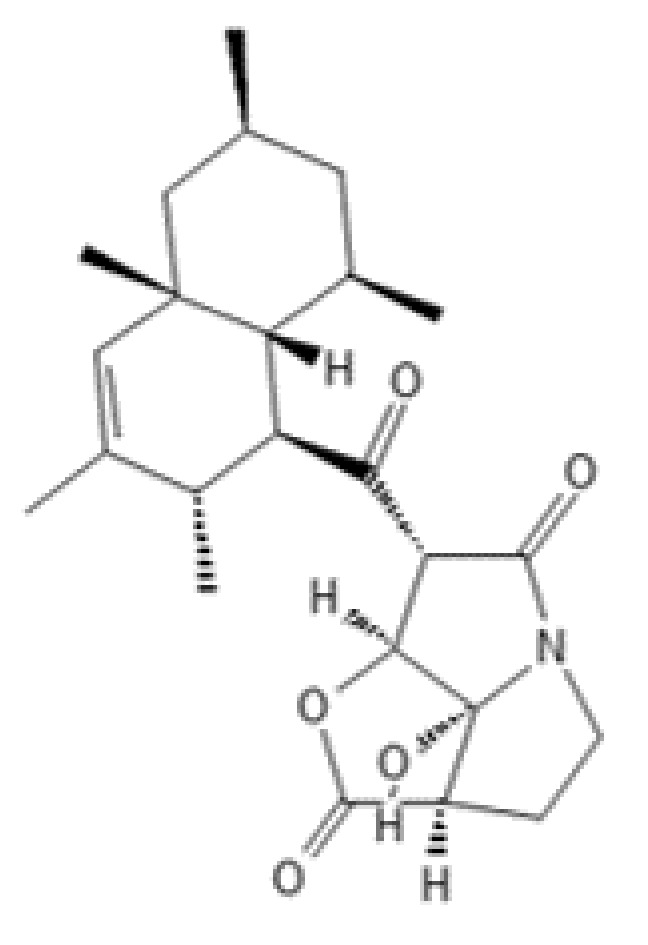
Structure of pyrrolizilactone.

In the course of phenotypic screening using the microbial metabolite fraction library for new anticancer compounds, a novel metabolite, pyrrolizilactone (Figure 10), was discovered and isolated from the culture broth of an uncharacterized fungus [129]. The newly identified compound (MF: C_24_H_33_NO_5_) has a unique structure: a tricyclic skeleton composed of a pyrrolizidinone moiety fused with a γ-lactone, which is connected to a decalin moiety by a ketone. Pyrrolizilactone has been reported to display moderate cytotoxic effects against human promyelocytic leukemia cell line HL60 and human cervical cancer cell line HeLa with IC_50_ values of 1.1 µg/mL (2.65 µM) and 3.1 µg/mL (7.46 µM), respectively.

Pyrrolizilactone has been classified as a proteasome inhibitor using two indirect target identification approaches—MorphoBase and ChemProteoBase profiling systems—based on specific changes in cellular morphology and on the intracellular proteome induced by chemical manipulations [130]. The target prediction has been confirmed both in cell-free and cell-based assays. Pyrrolizilactone markedly inhibited the trypsin-like activity of the human 20S proteasome with an IC_50_ value of 1.6 µM, as determined by in vitro enzymatic assays. The chymotrypsin-like and caspase-like activities were also inhibited by pyrrolizilactone in a dose-dependent manner, but to a lesser extent (IC_50_: 29 µM and 84 µM, respectively). Unlike conventional proteasome inhibitors that mainly target the β_5_ subunit/chymotrypsin-like site, pyrrolizilactone inhibits the trypsin-like activity of proteasomes. Treatment of HeLa cells with 30 µM pyrrolizilactone caused accumulation of ubiquitinated proteins, confirming its ability to inhibit 20S proteasomes within cells. Furthermore, pyrrolizilactone induced cell cycle arrest at G1 and G2/M in HeLa cells, as demonstrated by flow cytometric analysis [130].

### 2.9. Secondary Metabolites of Pestalotiopsis sydowiana

**Figure 11:**
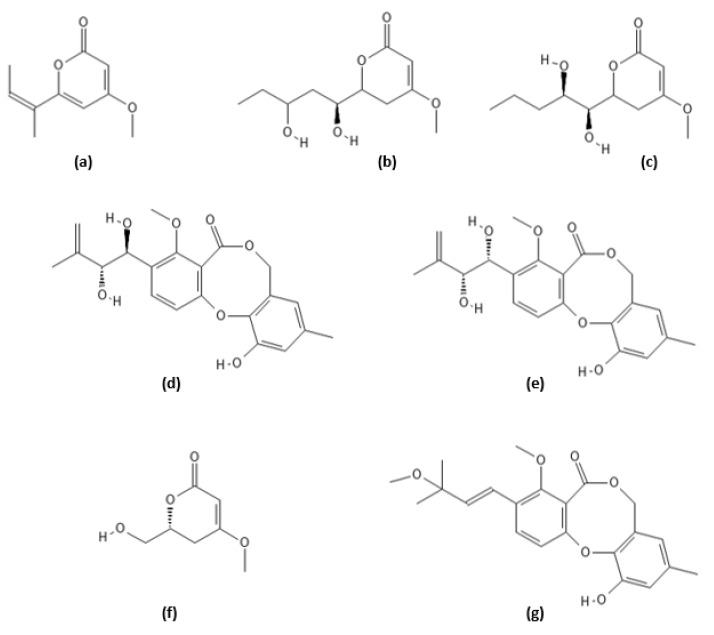
Structures of secondary metabolites of *Pestalotiopsis sydowiana*: (**a**) pestalotiopyrone G, (**b**) photipyrone B, (**c**) LL-P880β lactone, (**d**) pestalotiollide A, (**e**) pestalotiollide B, (**f**) 6-hydroxymethyl-4-methoxy-5,6-dihydro-2*H*-pyran-2-one, and (**g**) 3′-O-methyldehydroisopenicillide.

Endophytic fungi of the genus *Pestalotiopsis* are known to produce a variety of bioactive secondary metabolites with anticancer, antimicrobial, antifungal, and antioxidant activities [131,132]. In the course of screening for natural proteasome inhibitors, seven polyketides with proteasome inhibitory activity were isolated from the solid culture of *Pestalotiopsis sydowiana* from a rhizome of a halophyte, *Phragmites communis* Trinus [133]. Among them, three compounds have been identified as penicillide derivatives (3′-O-methyldehydroisopenicillide, pestalotiollide B, and pestalotiollide A [134]), and four compounds—as α-pyrone analogs (pestalotiopyrone G [135], LL-P880β lactone [136], photipyrone B [137], and 6-hydroxymethyl-4-methoxy-5,6-dihydro-2*H*-pyran-2-one) (Figure 11). The last metabolite was obtained from nature for the first time [133]. The isolated compounds have been found to inhibit the chymotrypsin-like activity of the human erythrocyte 20S proteasome in a dose-dependent manner. Pestalotiopyrone G (MF: C_10_H_12_O_3_) has demonstrated the most potent proteasome inhibitory activity with an IC_50_ value of 1.2 ± 0.3 µM. Photipyrone B and LL-P880β lactone have also inhibited the chymotrypsin–like activity of the 20S proteasome with IC_50_ values in a low micromolar range (7.9 ± 1.8 µM and 8.9 ± 1.5 µM, respectively). The other isolated polyketides have displayed their 20S inhibitory activity with IC_50_ of 12.4 ± 1.1 µM (pestalotiollide A), 18.5 ± 4.2 µM (pestalotiollide B), 20.5 ± 2.3 µM (6-hydroxymethyl-4-methoxy-5,6-dihydro-2*H*-pyran-2-one), and 30.5 ± 1.5 µM (3′-O-methyldehydroisopenicillide).

### 2.10. Higginsianin B

Bioactivity-guided fractionation of the mycelium extract of the ascomycete fungal pathogen *Colletotrichum higginsianum* has resulted in the isolation of two novel diterpenoid α-pyrones, named higginsianins A and B, with molecular formulas established as C_27_H_38_O_4_ and C_27_H_40_O_4_, respectively [138]. These compounds have been found to possess promising antiproliferative effects in a panel of six cancer cell lines including Hs683 (oligodendroglioma), U373 (glioblastoma), A549 (non-small-cell lung cancer), MCF-7 (breast carcinoma), SKMEL-28 (human melanoma), and B16F10 (murine melanoma) cell lines.

**Figure 12:**
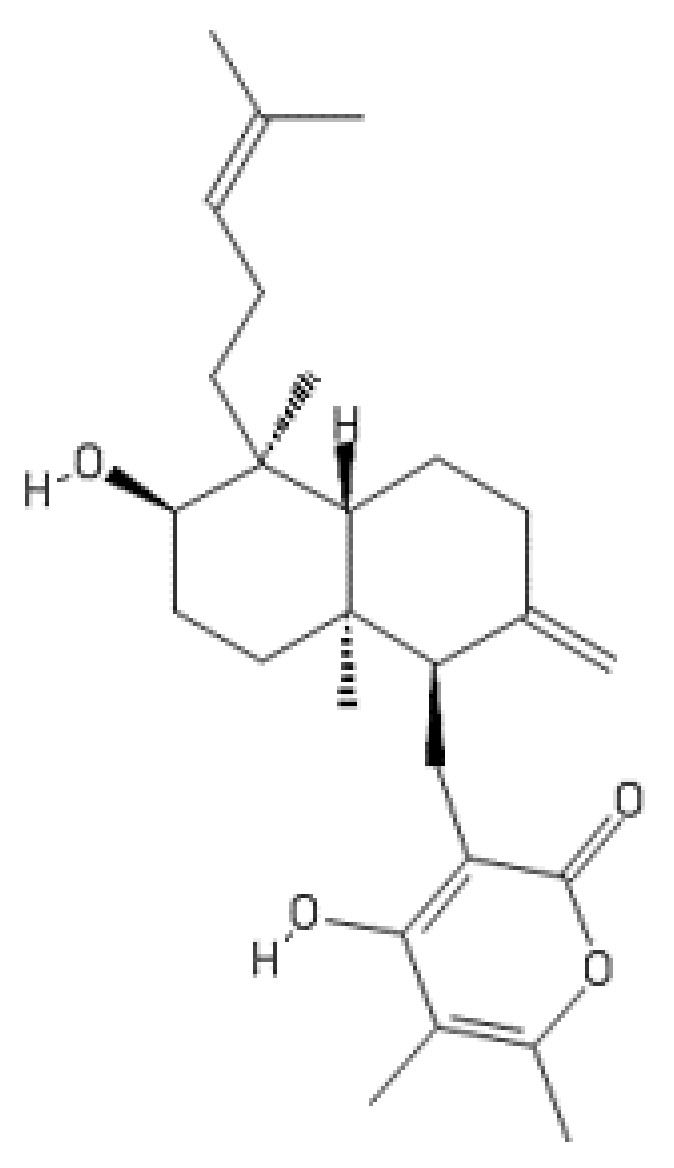
Structure of higginsianin B.

Higginsianin B (Figure 12) has recently been shown to inhibit human proteasome activity as demonstrated by in vitro enzymatic assays and cell-based assays [139]. The compound inhibited the chymotrypsin-like and caspase-like activities of the proteasome in a dose-dependent manner, with a maximal inhibition of around 40% reached at 5 µM. Cell-based assays have also demonstrated dose-dependent inhibition of chymotrypsin-like and caspase-like proteasomal activities in human diploid fibroblasts (BJ cells) exposed to higginsianin B at the concentration of 2.5–100 µM for 24 h and 48 h.

The higginsianin-producing fungus *C. higginsianum* causes anthracnose disease in numerous wild and cultivated members of the Brassicaceae, including *Arabidopsis thaliana*. Higginsianin B has been shown to suppress jasmonate-mediated plant defenses through inhibition of 26S proteasome-dependent degradation of JAZ proteins, the repressors of jasmonate responses [139].

## 3. Inhibitors of the E1 Ubiquitin-Activating Enzyme

### 3.1. Panepophenanthrin

**Figure 13:**
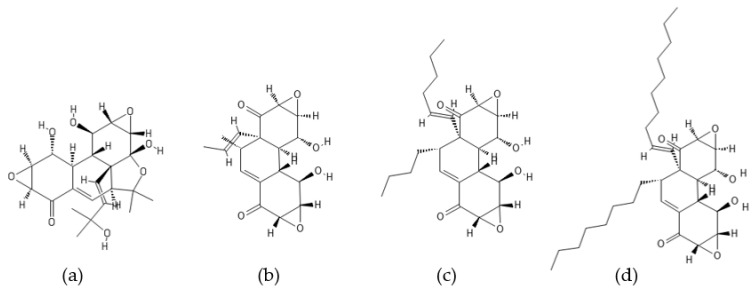
Structures of (**a**) panepophenanthrin, (**b**) RKTS-80, (**c**) RKTS-81, and (**d**) RKTS-82.

Panepophenanthrin, a dimeric epoxyquinone, was reported as the first natural product with inhibitory activity against the E1 ubiquitin-activating enzyme in the UPS [140]. Isolated from the fermentation broth of the basidiomycete fungus *Panus rudis* Fr. IFO 8994, panepophenanthrin (MF: C_22_H_28_O_8_) has a unique molecular structure that was determined by NMR and X-ray crystallographic analyses as 1,3a,10-trihydroxy-10c-(3-hydroxy-3-methylbut-1-enyl)-5,5-dimethyl-1,2,3,3a,5,5a,8,9,10,10a,10b,10c-dodecahydro-4-oxa-2,3,8,9-diepoxyacephenanthrylen-7-one (Figure 13) [140]. The complex structure of (+)-panepophenanthrin is characterized by a densely substituted tetracyclic skeleton, biosynthesis of which involves the Diels–Alder dimerization of two identical epoxyquinol monomers [141].

Panepophenanthrin has been found to inhibit formation of the E1–ubiquitin thioester intermediate in a dose-dependent manner with an IC_50_ value of 17.0 µg/mL (40.4 µM), as determined by immunoblot analyses using recombinant human E1 and biotinylated ubiquitin [140]. Although panepophenanthrin has inhibited binding of ubiquitin to the E1 enzyme in vitro, no significant inhibitory effect has been observed in intact cells up to the concentration of 50 µg/mL (119 µM). Because of its biological activity against the E1 ubiquitin-activating enzyme and unusual chemical structure, panepophenanthrin represents a highly attractive but challenging synthetic target. Despite these challenges, total syntheses of panepophenanthrin and its cell-permeable derivatives RKTS-80, RKTS-81, and RKTS-82 have been achieved [141,142,143]. These analogs, containing hydrophobic alkyl side chains, have been found to inhibit formation of the E1–ubiquitin complex much more potently than natural panepophenanthrin with IC_50_ values of 3.5 µM (RKTS-81), 9.4 µM (RKTS-80), and 90 µM (RKTS-82) [141]. Furthermore, RKTS-80-82 analogs blocked cell growth of human breast cancer MCF-7 cells in a dose-dependent manner with IC_50_ values of 1.0, 5.4, and 3.6 µM for RKTS-81, RKTS-80, and RKTS-82, respectively [141].

### 3.2. Himeic Acid A

**Figure 14:**
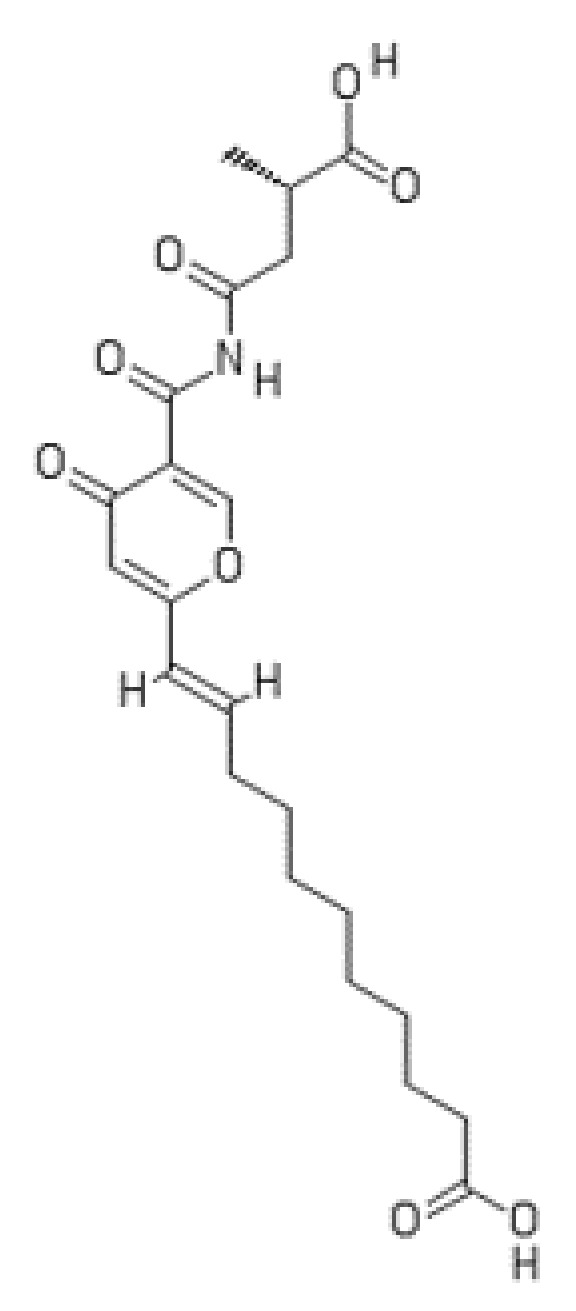
Structure of himeic acid A.

Himeic acid A is the second natural metabolite exhibiting inhibitory activity against the E1 ubiquitin-activating enzyme [144,145]. The compound was discovered in the course of a screening program for inhibitors of the UPS from natural sources for cancer therapy [64]. It was isolated from the mycelium of a marine-derived fungus, *Aspergillus* sp. (identified later as *A. japonicus* MF275 [145]) cultured in a fermentation medium [144]. The fungus was originally obtained from the mussel *Mytilus edulis* collected in Toyama Bay in the Sea of Japan. Marine-derived *Aspergillus* species have become important source of bioactive secondary metabolites [146]. Himeic acid A (MF: C_22_H_29_NO_8_) is a 2,5-disubstituted 4-pyronecompound with long fatty acyl and amide side chains (Figure 14). Biosynthesis of this natural product involves a polyketide synthase–non-ribosomal peptide synthase (PKS–NRPS) hybrid multienzyme complex [145].

Himeic acid A has been found to inhibit formation of the E1–ubiquitin intermediate by 65% at a concentration of 50 µM as determined by immunoblot analysis using FLAG-tagged human recombinant E1 and GST-ubiquitin [144]. Two cognate compounds isolated from *A. japonicus* MF275 along with himeic acid A, designated as himeic acids B and C, have shown only weak E1 inhibitory activity even at 100 µM [144]. Himeic acid A containing a 4-pyrone ring has been found to be nonenzymatically converted to himeic acid C, the corresponding 4-pyridone derivative, during the culture growth [147]. The production of himeic acid A has been highly pH-dependent. Since himeic acid A does not inhibit E1-like enzymes for other ubiquitin-like modifiers, namely, SUMO-1 and ISG15, it is considered to be a specific inhibitor of the ubiquitin E1 enzyme [145].

## 4. Inhibitors of E3 Ligases

Most ubiquitin protein ligases (E3s) such as RING domain-containing E3s (e.g., mouse double minute 2 (MDM2; HDM2 in humans)) lack any enzymatic activity. They act by bringing Ub-loaded E2 proteins into proximity with target proteins [21,22]. Thus, the inhibition of E3 ligases generally requires the targeting of protein–protein interactions. Overexpression of MDM2/HDM2, a dominant E3 ligase for the p53 tumor suppressor protein, has been observed in many types of cancer [148,149]. MDM2-mediated p53 ubiquitination can be inhibited by directly targeting the protein–protein interaction between p53 and MDM2 [150,151].

### 4.1. Chlorofusin

Chlorofusin was identified as the first natural product which antagonizes the p53–HDM2 interaction [152]. This metabolite was originally isolated from the fermentation broth of the microfungus *Fusarium* sp. 22026 (corrected as *Microdochium caespitosum* [153]) in the course of an activity-guided screening program involving the examination of over 53,000 microbial extracts. Purified chlorofusin has been found to disrupt the interaction between p53 and HDM2 at the micromolar level in a DELFIA-modified ELISA assay (IC_50_: 4.6 µM), but has shown no cytotoxicity against Hep G2 (human hepatocellular carcinoma) cells at 4 µM [152]. Surface plasmon resonance (SPR) spectroscopy revealed that the mode of action of chlorofusin, which antagonizes the p53–MDM2 interaction, involves direct binding to the N-terminal domain of MDM2 (K_D_ = 4.7 µM is in agreement with IC_50_: 4.6 µM) [153].

**Figure 15:**
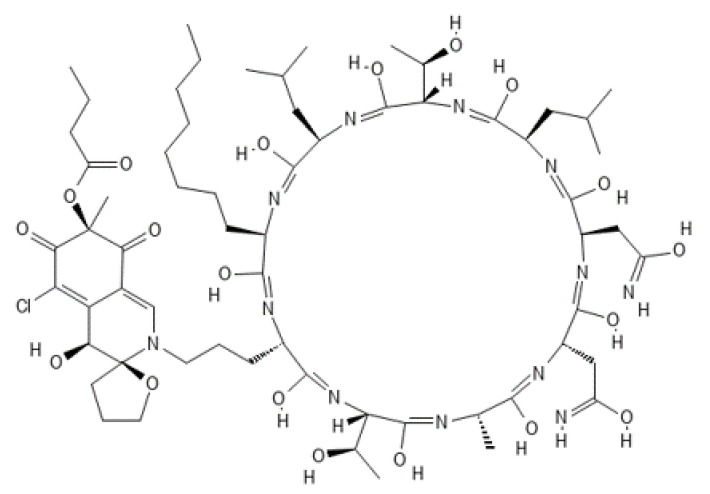
Structure of chlorofusin.

The molecular formula of chlorofusin has been identified as C_63_H_99_O_19_N_12_Cl. Its structure has been proposed to consist of a densely functionalized, azaphilone-derived chromophore linked through the terminal amine of ornithine to a cyclic peptide composed of nine amino acid residues (Figure 15) [152]. The absolute stereochemistry of the chromophore part has been determined and total syntheses of chlorofusin have been achieved [154,155,156,157]. Furthermore, chlorofusin analogs that have the ability to prevent the binding of p53 to HDM2 have been developed [158]. Two of these chlorofusin derivatives have been found to inhibit growth of SJSA-1 osteosarcoma cells. Studies of the natural product chlorofusin have also led to the identification of a new small-molecule inhibitor of the p53–HDM2 interaction [158].

### 4.2. Hexylitaconic Acid

**Figure 16:**
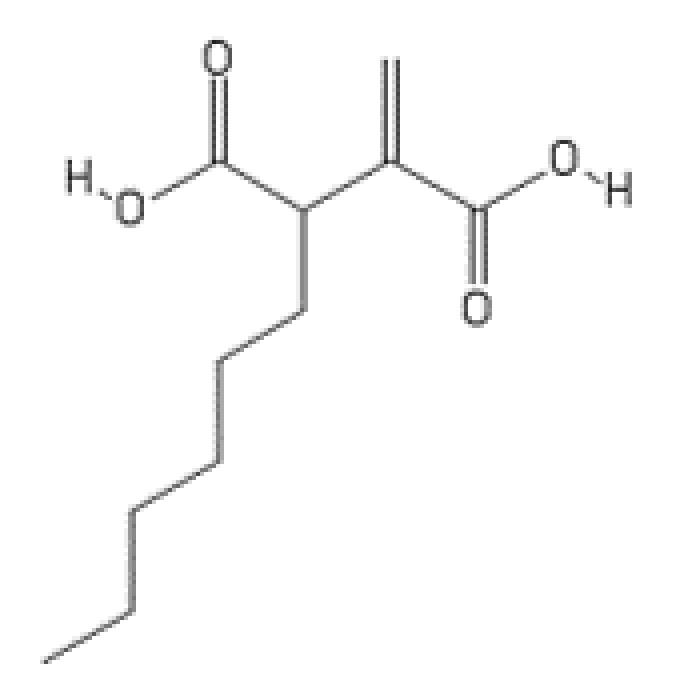
Structure of hexylitaconic acid.

Hexylitaconic acid (2-hexyl-3-methylensuccinic acid, MF: C_11_H_18_O_4_) (Figure 16) is the second natural product reported to inhibit the p53–HDM2 interaction [159]. Both enantiomers, (−) and (+), of this product have been found in nature; (+)-hexylitaconic acid was first isolated from the broth filtrate of *Aspergillus niger* strain K-88 (originally cultivated in field soil) and identified as a root growth stimulator of lettuce seedlings [160]. The (−) enantiomer was subsequently isolated as a secondary metabolite of the marine endophytic fungus *Apiospora montagnei* isolated from the alga *Polysiphonia violacea* [161]. Recently, two new dimeric hexylitaconic acids named asperwelwinates A and B were isolated from the marine-derived fungus *Aspergillus welwitschiae* CUGBMF180262 [162].

In the course of screening for natural inhibitors of the p53–HDM2 interaction, (–)-hexylitaconic acid was isolated from the culture medium of the marine-derived fungus *Arthrinium* sp. [159]. The fungus was originally separated from the marine sponge. The structure of the pure (−) enantiomer was identified by comparing its spectroscopic data with the values previously reported [159,161]. Inhibition of the p53–HDM2 interaction was evaluated by the ELISA method using purified recombinant p53 and HDM2 proteins and primary anti-MDM2 antibody [159]. The pure (−)-hexylitaconic acid from *Arthrinium* sp. has been found to inhibit the p53–HDM2 interaction in a dose-dependent manner with an IC_50_ value of 50 µg/mL (233 µM). The mode of action of (−)-hexylitaconic acid involves direct binding to HDM2 but not to the p53 protein since no inhibition of the p53–COP1 interaction has been observed [64,159]. The (+) enantiomer of hexylitaconic acid, prepared by means of chemical synthesis, has been reported to inhibit the p53–HDM2 interaction in a dose-dependent manner with an IC_50_ value comparable to that of the (−) enantiomer [163].

### 4.3. Potential Inhibitors of the MDM2–p53 Interaction

**Figure 17:**
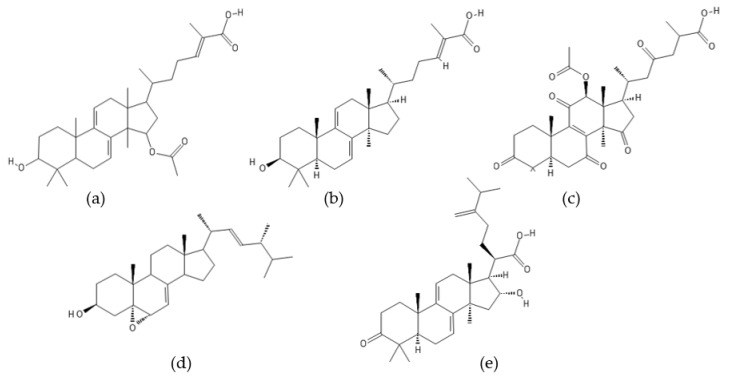
Structures of (**a**) ganoderic acid X, (**b**) ganoderic acid Y, (**c**) ganoderic acid F, (**d**) EMCD (5,6-epoxy-24(*R*)-methylcholesta-7,22-dien-3β-ol), and (**e**) polyporenic acid C.

The potential inhibitory activity against the p53–MDM2 interaction was virtually tested on a dataset of 40 low-molecular compounds previously isolated from mushrooms and described as having antitumor properties [164]. The docking tool AutoDock4 was used with known crystal structures of MDM2 obtained from the Protein Data Bank. This virtual screening has led to the identification of several steroids (Figure 17) as the most promising potential MDM2 inhibitors: ganoderic acid X (K_i_: 44 nM), ganoderic acid Y (K_i_: 47 nM), ganoderic acid F (K_i_: 59 nM), EMCD (5,6-epoxy-24(*R*)-methylcholesta-7,22-dien-3β-ol) (K_i_: 106 nM), and polyporenic acid C (K_i_ = 158 nM). Ganoderic acids X, Y, and F were originally isolated from the traditional Chinese medicinal mushroom *Ganoderma lucidum* and shown to be cytotoxic against some tumor cell lines, including human carcinoma cells [165]. Furthermore, ganoderic acid X has been found to induce apoptosis in human hepatoma HuH-7 cells [166]. EMCD, originally isolated from the medicinal fungus *Cordyceps sinensis*, has been reported to inhibit the proliferation of several tumor cell lines [167]. Another top-ranked potential MDM2 inhibitor, polyporenic acid C, was isolated from the mushroom *Piptoporus betulinus,* known as a source of anticancer agents [165,168]. It has been suggested that the antitumor activities observed for the top-ranked compounds may result from the disruption of the p53–MDM2 (HDM2) interaction and the consequent increase in p53 levels [164].

### 4.4. Statins as Inhibitors of the SCF^Skp2^ E3 Ligase

**Figure 18:**
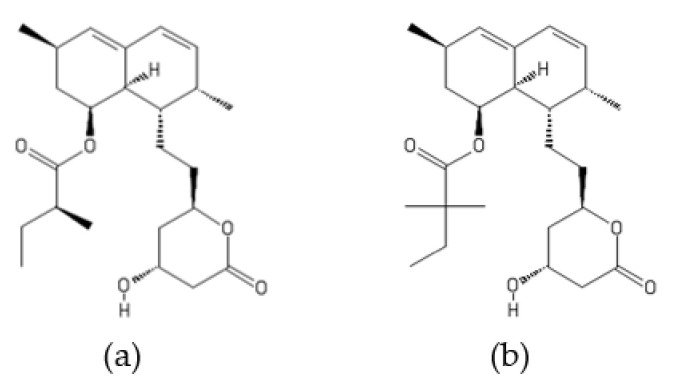
Structures of (**a**) lovastatin and (**b**) simvastatin.

Statins are a class of small fungal secondary metabolites approved as cholesterol-lowering drugs [169,170,171,172]. Their anticholesterolemic activity is due to their ability to inhibit 3-hydroxy-3-methylglutaryl-CoA (HMG-CoA) reductase, the essential enzyme in the cholesterol biosynthesis. The first statin discovered was mevastatin isolated from *Penicillium citrinum*. Later, lovastatin (formerly called mevinolin) was obtained from cultured broths of a soil fungus *Aspergillus terreus*. Recently, fruiting bodies of selected edible mushrooms have been reported as a potential source of lovastatin [173]. Simvastatin can be obtained by a semisynthetic process involving the chemical modification of the lovastatin side chain. Lovastatin and simvastatin (Figure 18) have also been reported to target proteasomes [174,175,176,177,178]. The prodrug forms of lovastatin and simvastatin contain a closed β-hydroxylactone ring, while the corresponding β-hydroxy acid is present in pharmacologically active drugs. Statins are converted from their prodrug (β-lactone) form to the active hydroxyl acid form in the liver where they inhibit the HMG-CoA reductase. However, it has been found that a small amount of the β-lactone form of statins remains in the body.

Lovastatin and simvastatin in their closed ring forms have been proposed to act as modulators of proteasomes since these statins have been found to stimulate the chymotrypsin-like activity of the purified 20S proteasome while inhibiting the caspase-like activity [174,175]. However, other studies have demonstrated inhibition of the chymotrypsin-like activity of proteasomes by statins in vitro and in vivo [176,177,178]. It has been reported that lovastatin and simvastatin in their prodrug form (β-lactone) inhibit the chymotrypsin-like activity of proteasomes, leading to the accumulation of cyclin-dependent kinase (CDK) inhibitors p21 and p27 (p21^Cip1^ and p27^Kip1^) and subsequent G1 arrest in breast cancer cell lines [176,177]. Simvastatin has also been found to inhibit proteasome activity in human non-small-cell lung cancer cells (A549 and NCI-H460) [178].

A large number of clinical and epidemiological studies indicate the potential of statins as repurposed drugs for the treatment of cancer [179,180]. It is important to note that studies over the past few years revealed that lovastatin and simvastatin can induce degradation of Skp2 (S-anaphase kinase-associated protein 2), a subunit of the SCF^Skp2^ ubiquitin ligase that targets p27^Kip1^ and p21^Cip1^ for proteasomal destruction [181,182]. The SCF^Skp2^ (Skp2-Skp1/Cullin-1/F-box-protein) complex is a multisubunit RING type E3 ligase, and the Skp2 F-box protein provides the substrate targeting specificity to the complex. The ligase SCF^Skp2^ is considered to be a promising target for cancer therapy since it selectively recognizes CDK inhibitors such as p27^Kip1^ and p21^Cip1^ for ubiquitination and subsequent degradation by the 26S proteasome [179,183].

## 5. Inhibitors of Deubiquitinases

Deubiquitinating enzymes (DUBs) play crucial roles in determining the cellular fates of many proteins. Since the polyubiquitin chain targets protein substrates to the 26S proteasome, most DUBs are natural antagonists of the proteasome. Because of the large number of potential DUB substrates and the exquisite specificity that some individual DUBs exhibit, the study of these enzymes as drug targets for therapeutic intervention has often been challenging [36,37,54,58,184,185].

### 5.1. USP7 Inhibitors

Ubiquitin-specific protease 7 (USP7) is a deubiquitinating enzyme that has activity on numerous cellular targets, including the oncogenic ubiquitin E3 ligase MDM2/HDM2 (described above in Section 4. Inhibitors of E3 Ligases). USP7 protects MDM2 from autoubiquitination and subsequent proteasomal degradation and thus downregulates the p53 tumor suppressor protein [36,37]. Therefore, USP7 is an attractive target for the development of therapeutics for cancer treatment [186,187,188].

#### 1′-(2-Phenylethylene)-ditryptophenaline

**Figure 19:**
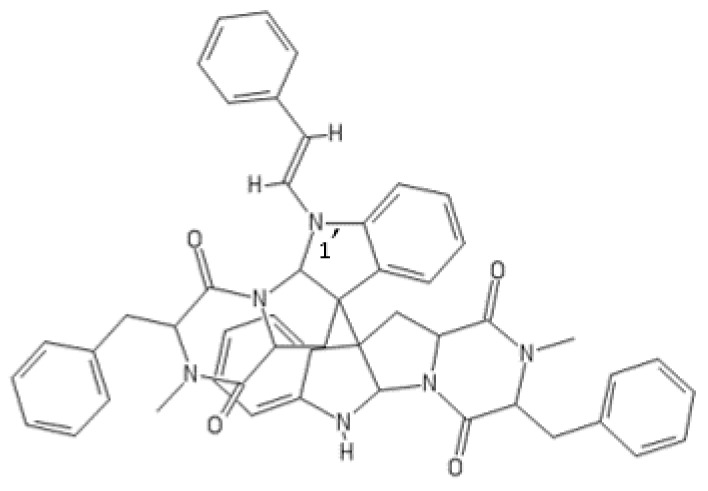
Structure of 1′-(2-phenylethylene)-ditryptophenaline.

The secondary metabolite 1′-(2-phenylethylene)-ditryptophenaline (MF: C_50_H_46_N_6_O_4_) (Figure 19) is a tryptophan-based homodimeric diketopiperazine alkaloid originally isolated from the culture extract of *Aspergillus flavu*s [189]. The fungus *A. flavus* is ubiquitous in nature and known to produce a wide spectrum of secondary metabolites [190]. Many dimeric diketopiperazine alkaloids exhibit a variety of biological activities and pharmacological effects [191].

In the course of bioactivity-guided screening of a series of synthetic tryptophan-based diketopiperazine alkaloids, 1′-(2-phenylethylene)-ditryptophenaline was identified as an inhibitor of ubiquitin-specific protease 7 (USP7) [192]. Among all the alkaloids tested, 1′-(2-phenylethylene)-ditryptophenaline has demonstrated the most potent USP7 inhibitory activity (90% inhibition at 10 µM). The presence of a phenylethylene group at the N-1′-position in the molecule of 1′-(2-phenylethylene)-ditryptophenaline is assumed to be important for USP7 inhibition because no inhibition has been observed for ditryptophenaline lacking the functional group.

### 5.2. Rpn11 Inhibitors

The Rpn11 (POH1 in humans) subunit of the lid subcomplex of the 19S regulatory particle is responsible for substrate deubiquitination during proteasomal degradation [30,31]. Rpn11 cleaves polyUb chains en bloc from substrates as they are degraded by proteasomes. Unlike all other known deubiquitinating enzymes (USPs, UCHs, OTUs, Josephins, and MINDY) that are cysteine proteases, Rpn11/POH1 is a Zn^2+^-dependent metalloprotease and contains a highly conserved Jab1/MPN domain-associated metalloisopeptidase (JAMM) motif [30,31].

#### Gliotoxin and Other Epidithiodiketopiperazines

Gliotoxin (Figure 6) is an epidithiodioxopiperazine (ETP)-type secondary metabolite that exhibits potent antibiotic and antitumor activities. Gliotoxin has been reported to act as a noncompetitive inhibitor of the chymotrypsin-like activity of proteasomes (as described above in Section 2.3. Proteasome Inhibitors). Anti-proteasome properties of gliotoxin have been attributed to its internal disulfide bond. However, recent studies using the polyubiquitinated protein substrate Ub^n^GST-Wbp2 (WW domain-binding protein 2, *n* > 30) to measure 26S proteasome-mediated protein degradation have revealed that gliotoxin and other ETPs inhibit proteasome activity in vitro and in cells by targeting the essential deubiquitinase Rpn11 [30,31] in the 19S regulatory complex of the 26S proteasome [111]. These molecules inhibit Rpn11 activity by chelating the catalytic Zn^2+^ ion [30,31]. Inhibition of the Rpn11 function by gliotoxin and its core scaffold compound, SOP11, results in proteasome malfunction and leads to cell death [111]. The ETP scaffold is a promising starting point to develop Rpn11 inhibitors, providing an alternative to achieving proteasome inhibition.

### 5.3. USP5/USP4 Inhibitors

Ubiquitin-specific proteinase 5 (USP5), also known as ubiquitin isopeptidase T (IsoT), specifically recognizes unanchored (not conjugated to target proteins) polyubiquitin chains and removes ubiquitin from the proximal end of the chain, which is essential for maintaining homeostasis of the free monoubiquitin pool [37,193]. Knockdown of USP5/IsoT leads to the accumulation of polyubiquitin and inhibition of proteasomal degradation due to competitive inhibition by the accumulated chains. Knockdown of USP5 has been found to inhibit proliferation of various cancer cell lines [37,193]. Since USP5 is overexpressed in cancer tissues, it can thus potentially serve as a new target for therapeutic interventions [194].

Ubiquitin-specific protease 4 (USP4), also known as a nuclear oncoprotein UNP (ubiquitous nuclear protein), removes monoubiquitinated and polyubiquitinated chains from its target proteins [195]. USP4 deubiquitinates many well-known proteins (e.g., tumor suppressors such as pRb) associated with the key processes involved in both cellular homeostasis and diseases, especially cancer, where USP4 is frequently overexpressed [195,196,197].

#### Vialinin A

**Figure 20:**
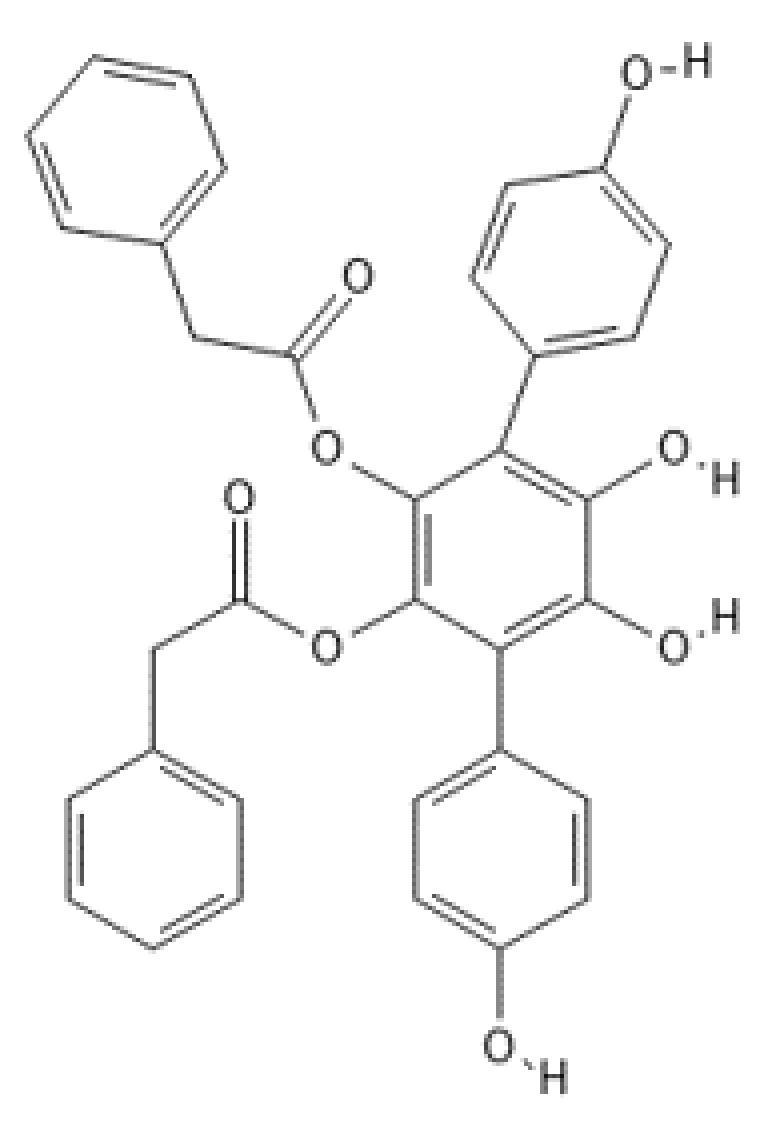
Structure of vialinin A.

Vialinin A (Figure 20) was originally isolated from dry fruiting bodies of the Chinese mushroom *Thelephora vialis* in the course of screening for new potent antioxidants from edible fungi and found to be a strong 2,3-diphenyl-1-picrylhydrazyl (DPPH) free radical scavenger [198]. Its chemical structure has been elucidated to be 5′,6′-bis(phenylacetoxy)-1,1′:4′,1”-terphenyl-2′,3′,4,4′’-tetraol (MF: C_34_H_26_O_8_) [198]. Vialinin A isolated from the other species belonging to the genus *Thelephora*, the symbiotic fungus *T. aurantiotincta*, has been reported to exhibit cytotoxicity against human hepatocellular carcinoma HepG2 cells and human colon carcinoma Caco-2 cells [199]. Vialinin A has also been found to exhibit effective anti-inflammatory activity by potently inhibiting TNF-α release from antigen-stimulated rat basophilic leukemia (RBL-2H3) cells (IC_50_: 0.09 nM) [200]. However, the compound has not inhibited TNF-α production in RBL-2H3 cells in a dose-dependent manner as effectively as TNF-a release from the cells.

In the search to elucidate the mode of action of vialinin A, ubiquitin-specific proteinase 5/isopeptidase T (USP5/IsoT) was identified as the molecular target of vialinin A by means of a beads probe method [201], and in another study, USP5 was reported to be one of the essential regulators of production of TNF-α [202]. Vialinin A has strongly inhibited the peptidase activity of USP5/IsoT with an IC_50_ value of 5.9 µM. In vitro ubiquitin-7-amido-4-methylcoumarin (Ub-AMC) hydrolysis assay also revealed inhibition of the activity of USP4 (IC_50_: 1.5 µM) and UCH-L1 (IC_50_: 22.3 µM) by vialinin A, but no significant inhibition has been observed for other deubiquitinases tested indicating that vialinin A is a semi-selective inhibitor for certain DUBs [201].

## 6. Conclusions

As the principal proteolytic pathway in eukaryotes, the ubiquitin–proteasome system plays a pivotal role in maintaining cellular proteostasis and regulates many basic cellular processes, including cell cycle progression, transcription, cell differentiation, apoptosis, signal transduction, morphogenesis, modulation of cell surface receptors and ion channels, antigen presentation, and protein quality control in the endoplasmic reticulum. For these reasons, it is not surprising that aberrations in the UPS are implicated in numerous human pathologies such as cancer, neurodegenerative disorders, autoimmunity, inflammation, or infectious diseases. Therefore, the UPS has emerged as an attractive target for drug discovery and development. For the past two decades, much research has been focused on identifying and developing both proteasome inhibitors and inhibitors of ubiquitinating/deubiquitinating enzymes. With the feature of unique structure and bioactivity, low-molecular-weight secondary metabolites serve as the lead compounds in the development of novel therapeutic drugs. Fungi produce a wide range of SMs which play a crucial role in fungal development, survival, and interactions with other organisms. Some fungal SMs were developed as essential medicines, such as antibiotics (e.g., penicillin) and cholesterol-lowering drugs (e.g., lovastatin). Recent studies have revealed potential of fungal SMs which may act as inhibitors of the UPS components. Re-emerging interest in fungal SMs as the lead structures in drug discovery requires methods to engineer biosynthesis of fungal SMs and activate BGCs that are often silent under conventional laboratory culture conditions. Recent advances in genome sequencing, bioinformatic tools, natural product chemistry, and an increasing ease in fungal genome manipulations have greatly enhanced the ability to efficiently mine fungal genomes for the discovery of new drugs and optimize secondary metabolite production [71,73,74,76,77,78,83]. The continued development and improvement of these approaches should allow the discovery of novel fungi-derived UPS inhibitors for therapeutic strategies.

## Figures and Tables

**Table 1:** Proteasome inhibitors.

Compound Name	Originally Derived from	Active Sites Targeted
Fellutamide B	*Penicillium fellutanum*	CHTL >> TL~CL
Fellutamide D	*Metulocladosporiella* sp.	CHTL^1^
Fellutamide E	*Metulocladosporiella* sp.	CHTL^1^
TMC-95A, TMC-95B	*Apiospora montagnei* Sacc. TC 1093	CHTL > CL > TL
TMC-95C	*Apiospora montagnei* Sacc. TC 1093	CHTL~ CL > TL
TMC-95D	*Apiospora montagnei* Sacc. TC 1093	CHTL > TL~CL
Gliotoxin	*Gliocladium fimbriatum*	CHTL > TL ≥ CL (Rpn11)^2^
Epoxyphomalin A	*Phoma* sp. (revised to *Paraconiothyrium* cf *sporulosum*)	CHTL~TL~CL
Epoxyphomalin B	*Phoma* sp. (revised to *Paraconiothyrium* cf *sporulosum*)	CHTL >> TL~CL
Neomacrophorins I, IV, VI, X	*Trichoderma* sp. 1212-03	CHTL~CL > TL
Trilongins BI–BIV	*Trichoderma longibrachiatum*	CHTL^1^
Terrein	*Aspergillus terreus* Thom	CHTL~TL^1^
Pyrrolizilactone	Uncharacterized fungus	TL >> CHTL > CL
Pestalotiopyrone G	*Pestalotiopsis sydowiana*	CHTL^1^
Photipyrone B	*Pestalotiopsis sydowiana*	CHTL^1^
LL-P880β lactone	*Pestalotiopsis sydowiana*	CHTL^1^
Pestalotiollides A, B	*Pestalotiopsis sydowiana*	CHTL^1^
6-hydroxymethyl-4-methoxy-5,6-dihydro-2*H*-pyran-2-one	*Pestalotiopsis sydowiana*	CHTL^1^
3′-O-methyldehydroisopenicillide	*Pestalotiopsis sydowiana*	CHTL^1^
Higginsianin B	*Colletotrichum higginsianum*	CHTL~CL^1^

^1^ Inhibition of TL and/or CL activities not determined. ^2^ See Table 4.

**Table 2:** Inhibitors of the E1 ubiquitin-activating enzyme.

Compound Name	Originally Derived from	Mechanism of Action
Panepophenanthrin	*Panus rudis* Fr. IFO 8994	Inhibits formation of the E1–ubiquitin thioester intermediate
Himeic acid A	*Aspergillus japonicus* MF275	Specific inhibitor of the E1 enzyme; inhibits formation of the E1–ubiquitin thioester intermediate

**Table 3:** Inhibitors of E3 ligases.

Compound Name	Originally Derived from	Mechanism of Action
Chlorofusin	*Fusarium* sp. 22026 (corrected as *Microdochium caespitosum*)	Antagonizes the p53–MDM2 interaction by direct binding to the N-terminal domain of MDM2
(–)-Hexylitaconic acid	*Apiospora montagnei*	Inhibits the p53–HDM2 interaction by direct binding to HDM2, but not to the p53 protein
(+)-Hexylitaconic acid	*Aspergillus niger* strain K-88	Inhibits the p53–HDM2 interaction
Ganoderic acids X, Y, F	*Ganoderma lucidum*	Potential inhibitors of the p53–MDM2 interaction
EMCD^1^	*Cordyceps sinensis*	Potential inhibitor of the p53–MDM2 interaction
Polyporenic acid C	*Piptoporus betulinus*	Potential inhibitor of the p53–MDM2 interaction
Lovastatin and simvastatin^2^	*Aspergillus terreus*	Induce the degradation of Skp2, a subunit of the SCF^Skp2^ ubiquitin ligase that targets p27^Kip1^ and p21^Cip1^ for proteasomal destruction

^1^ EMCD: 5,6-epoxy-24(*R*)-methylcholesta-7,22-dien-3β-ol. ^2^ Simvastatin can be obtained by means of a semisynthetic process involving the chemical modification of the lovastatin side chain.

**Table 4:** Inhibitors of deubiquitinases.

Compound Name	Originally Derived from	Cellular Target
1′-(2-phenylethylene)-ditryptophenaline	*Aspergillus flavu*s	USP7
Gliotoxin and other ETPs^1^	*Gliocladium fimbriatum*	Rpn11 (inhibition by chelating the catalytic Zn^2+^ ion)
Vialinin A	*Thelephora vialis*	USP5/IsoT, USP4

^1^ ETPs: epidithiodioxopiperazines.

## Data Availability

Not applicable.
